# Pregnant Patients with COVID-19 Admitted to an ICU: A Comparison with a Historical Cohort of Critical Pregnant Patients without COVID-19

**DOI:** 10.3390/life14020165

**Published:** 2024-01-23

**Authors:** Carmine Iacovazzo, Letizia Capasso, Carola Visani, Serena Salomè, Maria Vargas

**Affiliations:** 1 Department of Neurosciences, Reproductive and Odontostomatological Sciences, University of Naples Federico II, 80100 Naples, Italy; 2 Department of Translational Medical Sciences, Division of Neonatology, University of Naples Federico II, Via Pansini 5, 80131 Naples, Italy; letizia.capasso@unina.it (L.C.); serena.salome@unina.it (S.S.)

**Keywords:** maternal mortality, COVID-19, ICU

## Abstract

Aim of the study: Maternal mortality and ICU admissions have increased during the COVID-19 pandemic. We reported a case-series of pregnant patients with COVID-19 admitted to an ICU and we compared them with a historical cohort of pregnant patients admitted to an ICU without COVID-19. Methods: We included all pregnant patients with laboratory-confirmed COVID-19 infection admitted to our ICU in 2021. As a historical control group, we included all pregnant women who were admitted to our ICU between 1 January 2008 and 31 December 2013. Results: In 2021, 11 pregnant patients (pts) with COVID-19 were admitted to an ICU, representing 2.87% of ICU admissions. We found that pregnant patients with COVID-19 (1) had a higher BMI (34.6 vs. 28.8, *p* = 0.04) and a lower gestational age (30.6 vs. 34 weeks, *p* = 0.03), (2) were mainly admitted for respiratory failure (100% vs. 2.7%; *p* = 0.001) and (3) required more days of invasive and non-invasive ventilations (54.5% vs. 5.2%, *p* = 0.002), a longer duration of stay at the ICU (21.9 vs. 4.8 days, *p* < 0.0001) and had a higher mortality rate (27.3% vs. 0%, *p* = 0.0192). Conclusions: Pregnant patients with COVID-19 represent a challenge for ICU physicians due to their different characteristics and outcomes when compared to pregnant patients without COVID-19.

## 1. Introduction

The evidence on risk for adverse outcomes from coronavirus disease 2019 (COVID-19) among pregnant women is still emerging [1,2,3,4]. A documented COVID-19 diagnosis in obstetric patients was associated with multiple, concurrently documented adverse pregnancy outcomes, maternal complications and indicators of severe illness [1,2,3,4]. Maternal mortality rates have increased during the COVID-19 pandemic [1,2,3,4]. COVID-19 contributed to 25% of maternal deaths in 2020 and 2021 [2]. US maternal mortality rose more rapidly in 2021 than in 2020 and nearly doubled from the pre-pandemic rates in 2019 [2]. The rate of maternal deaths increased by 33.3% during the first 9 months of the COVID-19 pandemic, with the maternal death rate increasing by 74.2% among Hispanic individuals, 40.2% among non-Hispanic Black individuals, and 17.2% among non-Hispanic White individuals during the study period [3]. During the pandemic, the maternal death rate increased to 25.1 per 100,000 live births, up from 18.8 per 100,000 live births before the pandemic [4]. These findings suggest that the COVID-19 pandemic has had a negative impact on maternal health outcomes, with increased maternal mortality rates and disparities among racial and ethnic groups. Furthermore, during the pandemic, pregnant women with COVID-19 were commonly hospitalized and often required admission to intensive care units (ICUs) with ventilator support [5,6,7,8,9]. The prevalence of ICU admission among pregnant women with COVID-19 ranges from 13.3% to 30% [5,6]. One study found that almost all pregnant women admitted to an ICU with COVID-19 were unvaccinated [7]. Pregnant women with COVID-19 are also more likely to experience complications that can affect their pregnancy and developing baby compared to people without COVID-19 [7]. Risk factors for severe disease include being overweight or obese, >35 years old, having pre-existing comorbidity and being Black, Asian or a minority ethnicity [8]. It is important for pregnant women to lower the risk of contracting the disease, and to follow the guidelines of several clinical societies and local health authorities when caring for pregnant women with suspected or confirmed COVID-19 [9].

According to this, the aim of this brief report is to compare the mode of delivery, anesthesiologic procedures, characteristics and complications of the ICU stays of pregnant patients admitted to our ICU compared to those without the COVID-19 disease.

## 2. Materials and Methods

This observational study was performed at the University Hospital Federico II of Naples, Italy, a tertiary care facility with 3000 births per year and a referral center for high-risk pregnancies. The local ethic committee (Azienda Ospedaliero-Universitaria Policlinico di Federico II, Napoli. Ethic Committee, protocol number: 155/20) approved the investigative protocol, and written informed consent was obtained from each patient or next of kin. We included all pregnant patients with laboratory-confirmed COVID-19 infection admitted to our ICU in 2021. A comprehensive data collection was designed to include data about deliveries and their complication, anesthesia ASA class, reason of ICU admission, SAPS II, SOFA, duration of non-invasive and invasive ventilation, clinical features, treatments and complications and outcomes of ICU stays—newborn outcomes.

As a historical control group, we included all pregnant women that were admitted to the ICU between 1 January 2008 and 31 December 2013. Obstetric patients were identified from the ICU admission records. Characteristics of the patients admitted to the ICU were collected from the medical charts available in the archive of our department, recorded on a pre-filled form and entered in a computerized database using MS Office Excel 2007 (Microsoft, Redmond, WA, USA).

Data were reported as mean, range and standard deviation or percentage as appropriate. Non-parametric ANOVA or chi-squared tests were used for all the comparisons when appropriate. Statistical significance was set at 0.05. SPSS (IBM version 20) was used for the statistical analysis.

## 3. Results

In 2021, 11 pregnant patients with the COVID-19 disease were admitted to our ICU, representing 2.87% of our ICU admissions. The yearly percentage of pregnant patients without COVID-19 admitted to our ICU in the historical control group ranged from 0.8% to 2.5% (2008: 2.1%, 2009: 2.5%; 2010: 0.8%; 2011: 1.5%; 2012: 2%; 2013: 1.2%). There was no statistical significance in the rate of ICU admissions between pregnant patients with COVID-19 and pregnant patients without COVID-19, except for the 2010 (2.87% vs. 0.8%; *p* = 0.02). Table S1 showed the maternal characteristics at ICU admission. Pregnant patients with COVID-19 had a higher BMI (34.6 vs. 28.8, *p* = 0.04) and a lower gestational age (30.6 vs. 34 weeks, *p* = 0.03) than the pregnant patients without COVID-19. No differences were found in the comorbidities between the groups (Table S2). Patients in both groups delivered mainly with cesarean sections (100% vs. 95%). However, the pregnant patients with COVID-19 had a lower ASA classification compared to the pregnant patients without COVID-19 (ASA I and II 63.6% vs. 32%, *p* = 0.004; ASA III and IV 36.4% vs. 68%, *p* = 0.016). None of the pregnant patients with COVID-19 underwent hysterectomy, compared to the 45% of pregnant patients without COVID-19 (*p* = 0.002) that did. Table 1 shows the characteristics of the mode of delivery and anesthesiologic procedure in both groups. The pregnant patients with COVID-19 were mainly admitted to our ICU for respiratory failure (100% vs. 2.7%; *p* = 0.001), while the pregnant patients without COVID-19 were admitted for other reasons, such as severe post-partum hemorrhage or HELLP/AFLP (*p* = 0.07). Table 2 shows the characteristics of ICU admissions and complications during patients’ ICU stays. We did not find any differences in SAPS II and SOFA scores at patients’ ICU admissions, but the pregnant patients with COVID-19 required more days of invasive and non-invasive ventilations (10.7 vs. 1.4 days, *p* = 0.0041; 4 vs. 0.3 days, *p* < 0.0001, more vasoactive drugs (54.5% vs. 5.2%, *p* = 0.002), a longer ICU stay duration (21.9 vs. 4.8 days, *p* < 0.0001) and had a higher mortality rate (27.3% vs. 0%, *p* = 0.0192). Table S3 shows the clinical features and characteristics for each pregnant patient with COVID-19 admitted to our ICU. Moreover, the newborns of these patients needed more cardiopulmonary resuscitation maneuvers at birth (54.5% vs. 10%; *p* = 0.013). Table S4 reports the outcomes of newborns between the two groups. None of the newborns contracted COVID-19 at birth, and they were admitted to the neonatal ICU.

## 4. Discussion

This observational study aimed to compare the characteristics of pregnant patients with COVID-19 admitted to our ICU with a historical cohort of pregnant patients without COVID-19 admitted to our ICU in the previous years. Despite the ICU mortality being similar between the groups, around 2%, we found that the pregnant patients with COVID-19 (1) had a higher BMI and a lower gestational age at admission, (2) were mainly admitted for respiratory failure, (3) required more days of invasive and non-invasive ventilations, more vasoactive drugs and a longer ICU stay duration and that (4) newborns born at a lower gestational age had a greater need of cardiopulmonary resuscitation maneuvers at birth.

The ICU management of pregnant patients with COVID-19 is very complex. Admitting ICU care to pregnant patients means that the maternal and fetal status should be simultaneously taken into account. Our data showed that the characteristics and the evolutions of the hospitalization in the ICU within this group of patients were very different from the pregnant patients without the COVID-19 disease. According to this, the ICU’s management of the pregnant patients with COVID-19 required the experience of centers that specialized in pregnancy complications and respiratory diseases. Our university hospital is a tertiary care facility with more than 3000 births per year and a referral center for high-risk pregnancies [10]. Furthermore, it was previously involved in the Italian ECMO network for the use of ECMO in ARDS and it was the first regional hospital admitting COVID-19 patients at the beginning of the pandemic [11,12]. This identified a unique profile of expertise at our center in the regional and national fields. This paper may add more information about the clinical practice of pregnant patients with COVID-19 compared to non-COVID-19 pregnant patients. Contemporary literature about obstetrical COVID-19 patients falls is represented by epidemiologic reports depicting maternal and neonatal outcomes or case reports describing different clinical circumstances [11,12]. Although the effects of COVID-19 during pregnancy are deeply evaluated, data regarding severe and critical course of COVID-19 infections are still limited [13]. According to this, we were able to find reports comparing critical pregnant COVID-19 patients with non-pregnant COVID-19 or with non-critical COVID-19 patients [6,13].

Pregnant women with COVID-19 versus patients without COVID-19 are more likely to deliver preterm, and have an increased risk of maternal death and of being admitted to an intensive care unit [10]. In spite of this, the data about pregnant women suffering from severe COVID-19 and admitted to an ICU are sparse and the knowledge regarding this data is limited [14]. In a retrospective case series including pregnant patients with COVID-19 admitted to an ICU, the median gestational age at delivery was 36 weeks. Of the 38 postpartum patients, 14 (36.8%) were admitted to an ICU immediately following delivery due to their poor respiratory condition. In a recent report, postpartum patients were admitted to a critical care unit within 2 days after delivery in 75% of cases, and the cesarean section was chosen frequently at the time of delivery [15]. In our study, 54% of women were admitted to our ICU after the delivery, while 46 of them delivered two days before admission. However, in the future, the increasing knowledge on this topic may allow us to individualize the mode of delivery based on the following factors: maternal clinical status, fetal condition and obstetrical history [15].

Acute respiratory failure was the main reason for ICU admissions since COVID-19 in this kind of patients and mainly triggered respiratory symptoms. In a living systematic review, the ICU admission rate of pregnant women with COVID-19 is 3.4, while the same rate of pregnant woman without COVID-19 is 0.4 [16]. Our data about the ICU admission of pregnant patients with COVID-19 are in line with this. However, we found no differences in the comparison to the historical cohort of pregnant patients admitted to an ICU without COVID-19. In this report, critical pregnant patients with COVID-19 had a higher BMI. This is in line with the current literature; however, they had no pre-existing pulmonary or cardiac diseases that may have worsened their symptoms [13,17,18]. COVID-19 during pregnancy had a clinical course of respiratory disease. This is the reason why these patients required more days of mechanical ventilation and a longer ICU stay duration. Apart from this, critical pregnant patents without COVID-19 experienced more compilations that were strictly related to their gravidity. The need for mechanical ventilation was lower in patients admitted to the ICU in the antepartum period. However, the type of method used for delivery may improve maternal ventilatory parameters, facilitate proning, decrease maternal oxygen requirements and improve critical care outcomes [15].

In this report, we found that COVID-19 was associated with increased neonatal morbidity and complications; this was probably due to our patients having more preterm births. The timing of delivery in women admitted to an ICU due to severe COVID-19 was a matter of complying with the consensus guidelines suggesting to consider the delivery for women at <32 weeks of gestation only in the case of severe and refractory hypoxemia with a non-assuring fetal status [19]. Indeed, the gestational age at birth ranging from mother to mother with COVID-19 was the primary factor that affected the outcomes of neonates. Iatrogenic prematurity is the main cause of most neonatal complications, since the infection itself has a postnatal incidence ranging from 3% to 5% [19]. This may suggest that even the management of the newborns of pregnant patients with COVID-19 should be assisted in highly experienced facilities.

This study has several limitations. First, we were only able to include 11 pregnant patients with COVID-19 admitted to our ICU, so our results may not be applicable to the general population. Second, we obtained no data about viral loads or viral shedding. Third, our control group’s data referred back to a period from 2008 to 2013, and we had no recent data included in this study.

## 5. Conclusions

Pregnant patients with COVID-19 represent a challenge for ICU physicians due to having different characteristics and outcomes when compared to pregnant patients without COVID-19. Despite the ICU mortality being similar between these two groups, we found that pregnant patients with COVID-19 had a higher BMI and a lower gestational age at their ICU admission. Both mothers and newborns in the COVID-19 group needed more intensive care assistance. For this reason, we enquired about the management of these type of patients in referral centers for complicated pregnancy and respiratory diseases.

## Figures and Tables

**Table 1:** Mode of delivery and anesthesiologic procedures.

	With COVID-19 n = 11	*w*/*o* COVID-19 n = 38	*p*
Vaginal Delivery	0	2; 5%	0.432
Cesarean Section *	11; 100%	36; 95%	0.876
- Elective	0	3	0.819
- Planned	2	12	0.662
- Urgent	10	16	0.102
- Emergent	0	5	0.534
Spinal/Epidural Anesthesia	3; 36.4%	14; 37%	0.867
General Anesthesia	7; 63.6%	22; 63%	0.666
ASA I–II	7; 63.6%	12; 32%	0.004
ASA III–IV	4; 36.4%	26; 68%	0.016
ASA V	0	0	-
Hysterectomy	0	17; 45%	0.002
Uterine Artery Embolization	0	3; 8%	0.085

* According to Lucas’s classification of urgency of cesarean section.

**Table 2:** Characteristics of ICU admission and complications during ICU stay.

	With COVID-19 n = 11	*w*/*o* COVID-19 n = 38	*p*
ICU admission before delivery	5/11; 45.%	0/38; 0%	0.06
ICU admission after delivery	6/11; 54.4%	38/38; 100%	0.171
Intubation at ICU admission	8/11; 72.7%	25/38; 65.7%	0.843
Reason for ICU admission			
- Severe post-partum hemorrhage	0	19/38; 50%	0.082
- Puerperal sepsis	0	1/38; 2.6%	0.352
- Eclamptic/preeclamptic disease	0	7/38; 18.4%	0.548
- HELLP/AFLP	0	10/38; 26.4%	0.069
- Respiratory failure	11/11; 100%	1/38; 2.6%	0.001
SAPS II (points)	26.2 (5.7)	27.7 (12.2)	0.058
SOFA (points)	3.9 (1.1)	5.4 (2.73)	0.076
Days of invasive ventilation (days)	10.7 (18.9)	1.4 (2.8)	0.004
Days of non-invasive ventilation (days)	4 (2)	0.3 (1)	<0.0001
Vasoactive drugs	6/11; 54.4%	2/38; 5.2%	0.002
Extracorporeal depuration techniques	2/11; 18.2%	2/38; 5.2%	0.209
Complications during ICU stay			
- Bleeding from surgical site	0	4/38; 10.5%	0.890
- Neurologic disease (PRES, ischemia, hemorrhage)	1/11; 9%	6/38; 15.7%	0.232
- Others	0	0	-
ICU length of stay (days)	21.9 (21.6)	4.8 (3.9)	<0.0001
ICU mortality	3/11; 27.7%	0/38	0.019

## Data Availability

Data are available from the corresponding author after a motivated request.

## Supplementary Materials

The following supporting information can be downloaded at: https://www.mdpi.com/article/10.3390/life14020165/s1, Table S1: Maternal characteristics at the ICU admission. Table S2: Maternal comorbidities of the patients at the ICU admission. Table S3: clinical features and characteristics of pregnant patients with COVID-19 admitted in ICU. Table S4: newborn outcomes between the groups.
