# Supplementary material for: Pregnant Patients with COVID-19 Admitted to an ICU: A Comparison with a Historical Cohort of Critical Pregnant Patients without COVID-19
Source: Life (Basel). 2024 Jan 23;14(2):165. doi: 10.3390/life14020165 (PMC10890200; doi:10.3390/life14020165)
Supplement: Supplementary file 1 [file life-14-00165-s001.zip › life-2694435-supplementary.pdf]

Pregnant Patients with COVID-19 Admitted to an ICU: A Comparison with a Historical Cohort of Critical Pregnant Patients without COVID-19

Carmine Iacovazzo [1], Letizia Capasso [2], Carola Visani [1], Serena Salomè [2] and Maria Vargas [1,*]

[1] Department of Neurosciences, Reproductive and Odontostomatological Sciences, University of Naples Federico II, 80100 Naples, Italy
[2] Department of Translational Medical Sciences, Division of Neonatology, University of Naples Federico II, Via Pansini 5, 80131 Naples, Italy; letizia.capasso@unina.it (L.C.); serena.salome@unina.it (S.S.)
* Correspondence: vargas.maria82@gmail.com; Tel.: +39-0817473550

Table S1. Maternal characteristics at the ICU admission.

	**Pregnant pts with COVID-19 n = 11**	**Pregnant pts without COVID-19 n = 38**	***p***
Maternal age (y) (mean; SD; range)	32.2; 4.6; 25–40	32.9; 6.3; 18–44	0.77
Body mass index (kg/m^2) (mean; SD; range)	34.6; 7.7; 24.91–47.75	28.8; 4.2; 22.3–36.4	0.04
Primigravida	2; 16.6%	12; 31%	0.13
Patients with at least one previous cesarean section (n; %)	8; 73%	22; 58%	0.65
Multiple pregnancy ** (n; %)	1; 8.4%	2; 5%	0.45

Table S2. Maternal comorbidities of the patients at the ICU admission.

**Comorbidities**	**Pregnant pts with COVID-19 n = 11**	**Pregnant pts without COVID-19 n = 38**	
Heart disease	0%	5; 13%	0.21
Arterial Hypertension	0	2; 3%	0.45
Hemostatic disorder	0	1; 5%	0.6
Asthma	0	1; 2.5%	0.61
Autoimmune disease	0	1; 2.5%	0.58
Hypothyroidism	2; 20%	3; 8%	0.26
Malignancy	0	2; 5%	0.42
Chronic renal failure	0	0	-
Diabetes mellitus type I	0	1; 2.5%	0.78
Chronic Infection (HIV, HBV)	0	1; 2.5%	0.78
Cerebrovascular accident	0	2; 5%	0.67
Twin pregnancy	1; 9%	2; 5%	0.9

Table S3. clinical features and characteristics of pregnant patients with COVID-19 admitted in ICU.

Case	Vaccinal status	Gestational age at ICU Admission (*weaks*)	Delivery during Admission	Days between Admission and Delivery	Age at Gestational Delivery (*weaks*)	Mode of Delivery	Indication for Delivery	Pao /FIO before Intubation	Duration of Mechanical Ventilation† (*d*)	ICU Length of Stay† (*d*)	Hospital Length of Stay† (*d*)	Maternal Outcome	CT COVID-19 score (according Chung - Radiology 2020; 295:202-207)
1	No	26	yes	3	27	Cesarean section	Respiratory failure	71.9	45	60	60	Alive	19/20
2	No	26	yes	1	26	Cesarean section	Respiratory failure	46.5	26	46	55	Alive	19/20
3	no	27	No (delivery the day before the admission	0	27	Cesarean section	Respiratory failure	75.7	56	56	67	Dead	18/20
4	No	36	No (delivery 2 days before admission)	0	35+5	Cesarean section	Respiratory failure	39.7	6	6	12	Dead	15/20
5	No	38	Yes	1	38+1	Cesarean section	Respiratory failure	83.7	4	6	21	Alive	18/20
6	No	33	No (4 days before admission)	4	33+3	Cesarean section	Respiratory failure	Only non-invasive ventilation	2	3	7	Alive	14/20
7	No	33	No (2 days before admission)	2	33	Cesarean section	Respiratory failure	Only non-invasive ventilation	2	5	9	Alive	11/20
8	No	33	Yes	0	33	Cesarean section	Respiratory failure	98	10	16	21	Alive	17/20
9	No	30	Yes	0	30+1	Cesarean section	Respiratory failure	56	3	6	11	Alive	12/20
10	No	33	No (1 day before admission)	2	33+1	Cesarean section	Respiratory failure	91.5	13	14	16	Dead	18/20
11	No	19	No	6	20	Cesarean section	Status epilepticus	87	17	23	51	Alive	5/20

Table S4. newborn outcomes between the groups.

	**Newborn from Pregnant pts with COVID-19**	**Newborn from Pregnant pts without COVID-19**	***p***
Gestational age (weeks)	30.6; 5.4	34; 4	0.03
Born alive	11/12	36/40	0.96
Birth weight (gr)	1754.54; 770.6	2328.15; 841.3	0.048
Apgar at 5 minutes	7.6; 1.6	7.94; 1.61	0.43
Cardiopulmonary resuscitation at the birth	6/11	4/40	0.013
